# Molecular Cloning and Characterization of a Surface-Localized Adhesion Protein in *Mycoplasma bovis* Hubei-1 Strain

**DOI:** 10.1371/journal.pone.0069644

**Published:** 2013-07-23

**Authors:** Xiaohui Zou, Yuan Li, Yang Wang, Yumei Zhou, Yang Liu, Jiuqing Xin

**Affiliations:** 1 National Contagious Bovine Pleuropneumonia Reference Laboratory, Division of Bacterial Diseases, State Key Laboratory of Veterinary Biotechnology, Harbin Veterinary Research Institute, CAAS, Harbin, China; 2 National Engineering Research Center of Veterinary Biologics, Harbin, China; Miami University, United States of America

## Abstract

*Mycoplasma bovis* (*M. bovis*) is an important pathogen that causes various bovine diseases, such as mastitis in cows and pneumonia in calves. The surface proteins are generally thought to play a central role in the pathogenesis of this organism. We screened the entire genome of *M. bovis* Hubei-1 and discovered a gene named *vpmaX* that encodes the 25 kDa variable surface lipoprotein A (VpmaX). Sequence analysis revealed that VpmaX contains several repetitive units and a typical bacterial lipoprotein signal sequence. The *vpmaX* gene was cloned and expressed in *E. coli* to obtain recombinant VpmaX (rVpmaX). Western blot analysis using a rabbit antibody against rVpmaX demonstrated that VpmaX is a membrane protein. Immunostaining visualized via confocal laser scanning microscopy showed that rVpmaX was able to adhere to embryonic bovine lung cells (EBL), and this was also confirmed by a sandwich ELISA. In summary, a surface-localized adhesion protein was identified in *M. bovis* Hubei-1.

## Introduction

Usually regarded as the smallest known self-replicating organisms, mycoplasmas are widely distributed among a huge range of hosts, and many are infectious agents of animals and humans. *Mycoplasma bovis* (*M. bovis*) was first isolated in the United States in 1961 [1] and can lead to persistent respiratory disorders, arthritis, and tenosynovitis in feedlot cattle, as well as mastitis, otitis media, and keratoconjunctivitis in younger calves [2], [3]. Although many efforts have been taken to explore its pathogenesis, little progress has been made because this organism’s lack of a cell-wall renders traditional genetic tools used in classical bacterial agents ineffective in Mollicutes [4].

Many researchers have shown that the surface lipoproteins of mycoplasma play an important role in infection and organ lesion [5]. Lipoproteins have been shown to function in apoptosis [6], ABC transporter operation [7], strain virulence diversity [8], [9], and cytoadhesion [10], [11]. The family of variable surface lipoproteins (Vsps) in *M. bovis* PG45 and HB0801 has attracted the most research interest in recent studies, and some members in this family, such as VspA, VspB, VspE, and VspF, were successfully proved to possess adhesion ability [11]. However, a previous study demonstrated that *vsp* gene cluster was deleted in the *M. bovis* strain Hubei-1 [12].

Because adhesion to the host cell is a prerequisite for the colonization and infection of the host, the identification of adhesion proteins in pathogens is important for understanding the mechanisms of its pathogenesis. Several surface proteins and lipoproteins in mycoplasmas have been identified and implicated to play roles in cell adherence: the P1 and P30 proteins of *Mycoplasma pneumonia* [13], [14], the Maa1 and Maa2 proteins of *Mycoplasma arthritidis* [15], the GapA and CrmA proteins of *Mycoplasma gallisepticum* [16]. In *M. bovis*, several putative adherence proteins were identified, such as P26 [17], α-enolase [18], and members of the Vsps family [11].

Previous reports that investigated the whole genome of *M. bovis* [19], [20] allowed for the identification of adhesion proteins in this organism. *M. bovis* infection is increasingly pervasive in China, and the strain *M. bovis* Hubei-1 was first isolated in the Hubei province of China [21]. A previous report demonstrated that this strain was able to adhere to embryonic bovine lung (EBL) cells, even despite the absence of the *vsp* gene cluster in its genome; this implies that other adhesion proteins exist in the Hubei-1 strain. Our lab has reported that a surface-located α-enolase is an adhesion-related protein in *M. bovis* Hubei-1 [18]. Here, we analyzed the entire *M. bovis* Hubei-1 genome [20], and we identified the gene *vpmaX* (GenBank: AEI90145.1) that encodes a protein named “variable surface lipoprotein A” (VpmaX) according to GenBank. However, it is absolutely different from the VspA protein in PG45 (GenBank: ADR25410.1). Our report aims to characterize Hubei-1 *vpmaX* and the adhesion ability of its encoded protein.

## Materials and Methods

### Ethics Statement

All of the animal experiments were conducted under the supervision of the Harbin Veterinary Research Institute of the Chinese Academy of Agricultural Sciences in accordance with animal ethics guidelines and approved protocols. The Harbin Veterinary Research Institute Animal Ethics Committee approval number was SYXK(Hei) 2011–022.

### Computer Analysis of DNA Sequence and Protein Structure

The protein and DNA sequences were aligned with Needle (v6.0.1). Repetitive domains and transmembrane regions within VpmaX were detected using Dotlet (http://myhits.isb-sib.ch/cgi-bin/dotlet) and SOSUI (http://bp.nuap.nagoya-u.ac.jp/sosui/sosui_submit.html), respectively.

### Mycoplasma Strain, Cell Line, and Culture

Mycoplasma was cultured in modified pleuropneumonia-like organism (PPLO) medium supplemented with 20% inactivated horse serum (Hyclone, Logan, WV, USA), 10% yeast extract, thallium acetate (0.125 mg/ml) and penicillin (200 IU/ml). The origin and growth conditions of EBL cells have been described previously [18].

### Expression and Purification of Recombinant VpmaX

The *vpmaX* open reading frame was amplified by PCR using primer F (5′-cag gga tcc atc aat aaa ttg cta ata tct gct gt-3′) and primer R (5′-cag gtc gac tta aat ttt ctc aaa tat tgg tct aag-3′), subcloned into the vector pET-28a(+) and expressed in *E. coli* DE3 cells (Novagen, Madison, WI, USA). His-tagged proteins were purified by nickel affinity chromatography (Thermo, Rockford, IL, USA). The purified recombinant proteins were analyzed by electrophoresis on sodium dodecyl sulfate (SDS)-12% polyacrylamide gels (12% SDS-PAGE).

### Production of Anti-rVpmaX Immune Serum

Monospecific antiserum to a purified fusion protein was raised in female New Zealand White rabbits. The pre-immune serum was collected as a negative control, followed by intramuscular immunization on day 1 with 500 µg recombinant protein mixed with an equal volume of Freund’s complete adjuvant. Two subsequent immunizations with equal amounts of protein in Freund’s incomplete adjuvant were implemented at 2-week intervals. The antibodies were purified from the antisera and quantified according to previously reported methods [18].

### Immunoblot and Cellular Localization of VpmaX in *M. bovis* Hubei-1

The methods used to determine the localization of VpmaX in *M. bovis* was described in a previous report from this laboratory [18]. Briefly, *M. bovis* membrane and cytosolic proteins were separated with a ProteoExtract Transmembrane Protein Extraction Kit (Novagen) according to the manufacturer’s instructions. The proteins from the two protein fractions were separated by 12% SDS-PAGE and transferred to a nitrocellulose membrane (PALL, Ann Arbor, MI, USA). After blocking with 5% gelatin for 2 h at 37°C, the nitrocellulose (NC) membranes were incubated for 1 h at 37°C in a 1∶100 dilution of rabbit anti-rVpmaX serum in 5% gelatin. The membranes were then vigorously washed three times for 10 min each in PBS containing 0.05% Tween 20 (PBST) and incubated for 1 h at 37°C with a 1∶8000 goat anti-rabbit IgG (whole-molecule) peroxidase conjugate (Sigma). After three washings, the reactive protein bands on the membrane were visualized with a DAB Substrate Kit (Thermo). Bovine serum albumin and rVpmaX served as the negative and positive controls, respectively.

### Direct Adhesion of rVpmaX to EBL Cells and Adhesion Inhibition

To test the ability of rVpmaX to adhere to EBL cells, confocal laser scanning microscopy (CLSM) was used to observe the direct adhesion of rVpmaX to the EBL cell surface. EBL cells were propagated in a cell culture dish for 24–36 hours. The cells were washed twice and fixed in 4% PBS-buffered paraformaldehyde (Sigma) for 30 min at room temperature. The fixed cells were then blocked with PBS containing 1% (wt/vol) bovine serum albumin (PBS-BSA) for 2 h at 37°C. After washing, the cells were incubated in 1 ml PBST containing 10 or 20 µg rVpmaX for 1 h at 37°C. To ensure that the protein was functionally active, the rVpmaX was used as soon as possible after it was purified. Cells incubated with PBST without protein served as a negative control. In the adhesion inhibition test, the protein was incubated with 10 or 20 µl of rabbit anti-rVpmaX serum in 1 ml PBST for 30 min at 37°C before adding it to the fixed cells. The excess protein was removed by successive washes, and 1 ml of a 1∶100 dilution of rabbit anti-rVpmaX serum in PBS-BSA was added to all of the dishes (including the control dish) and stored at 4°C overnight. After washings, the dishes containing the cells were overlaid with a 1∶100 dilution of goat anti-rabbit IgG (whole molecule)–FITC (Sigma) at 37°C for 1 h to stain the protein adhered to the EBL cells. The cell membranes and nuclei were labeled according to methods described previously [17]. The immunofluorescence was detected using a Leica TCS SP5 laser scanning confocal microscope (Leica, Mannheim, Germany).

### Sandwich ELISA

To determine the specificity of rVpmaX adhesion to EBL cells and quantify the adhesion process, we devised a sandwich ELISA by modifying a previous method [22]. A flat-bottom 96-well ELISA plate (Jet Biofil, Guangzhou, China) was coated with EBL cell membrane or cytosolic protein fractions and blocked with bovine serum albumin in PBS (PBS-BSA). For the adhesion assay, the rVpmaX protein was serially diluted with PBST and applied to either coated or blank plates. For the adhesion inhibition assay, rVpmaX was incubated with rabbit anti-rVpmaX serum at different dilutions before interacting with the coated proteins. After incubating for 1 h at 37°C, unbound proteins were removed by washing, and the interaction between proteins was evaluated by adding rabbit anti-rVpmaX serum and goat anti-rabbit IgG peroxidase conjugate (Sigma). The absorbance was read at 450 nm in an ELISA plate reader (Bio-Tek, Winooski, VT, USA). The test was repeated three times. The rVpmaX was replaced with the equivalent *M. bovis* membrane fraction proteins in additional adhesion and adhesion inhibition assays to confirm adhesion ability of natural VpmaX.

## Results

### Structural Features of *vpmaX* and its Deduced Protein

There are two distinct repetitive units that begin at the N-terminal and extend 100 aa towards the C-terminal. The longer reiterated sequence, KPSEQGSGTNSQQGSG,is directly repeated 3 times, and the shorter repetitive unit, QGSG, is repeated 7 times. While each of the former long repetitive unit contains two latter short repeats (QGSG). Blasting these two reiterated units against mycoplasma genomes reveals that the two repetitive sequences repeat only in VpmaX. A comparison of the *vsp* genes in *M. bovis* HB0801 and PG45 reveals that the 71-bp region upstream of the ATG initiation codon contains a putative ribosome binding site and exhibits 99% homology among all *vsp* genes [12], [23]. The region upstream of *vpmaX* shows only 48.7% identity to the same region in the *vsp* genes in PG45 and HB0801. The Vsp N-terminal region containing 31 aa is also a highly conserved domain that displays 99% amino acid identity among the Vsps in *M. bovis* HB0801 and PG45 [12], [23]. However, there was only a 40.6% identity in this area between VpmaX and Vsps in HB0801 and PG45. Another structural characteristic was the high serine (12.2%) and lysine (11.8%) content of VpmaX; in PG45 VspA, VspB, and VspE, the abundant amino acid was proline [24].

Although VpmaX possesses a different N-terminal sequence than Vsps from PG45, its N-terminal region is consistent with the typical structure of a prokaryotic prolipoprotein signal peptide [23], [25]. It begins with two leucine and isoleucine residues (three leucines in Vsps of PG45) followed by a core of 20 hydrophobic aa and terminates with the tetrapeptide Ala-Ala-Ser-Cys. An analysis of the amino acid sequences with SOSUI also predicted a transmembrane region in the N-terminal of VpmaX.

Interestingly, the *M. bovis* strain HB0801, which was also isolated from Hubei province, contains a protein named variable lipoprotein VspX (GenBank: AFM51825.1) that has a protein sequence 100% identical to *M. bovis* Hubei-1 VpmaX. The most similar protein identified in the *M. bovis* PG45 genome was a putative lipoprotein (PL) (GenBank: ADR24803.1) of 195 aa that had an 81% identity with VpmaX. Further investigation revealed that 25 aa in the C-terminal of VpmaX was deleted in PL, the fifth QGSG repetitive units of VpmaX was removed, and the third and sixth QGSG units were replaced by other sequences in *M. bovis* PG45 PL.

### Expression of Recombinant *M. bovis* VpmaX in *E. coli*

**Figure 1:**
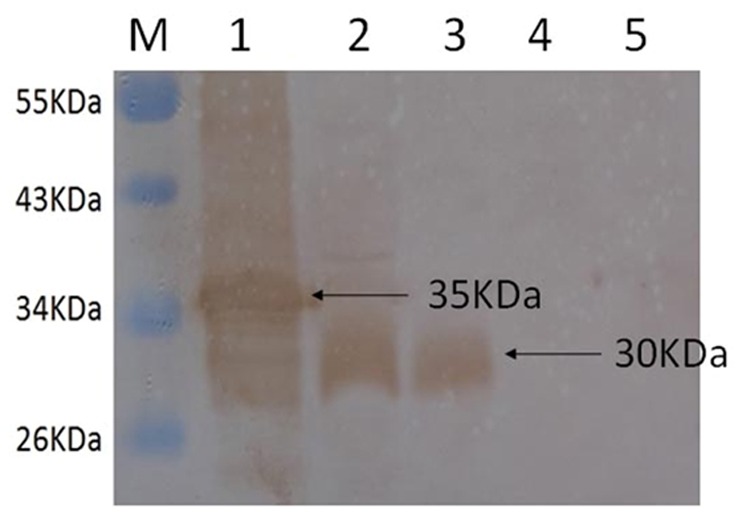
Localization of VpmaX in *M. bovis* Hubei-1. Western blot analysis of rVpmaX (lane 1), *M. bovis* total proteins (lane 2), cell membrane fraction proteins (lane 3), cell soluble cytosolic fraction proteins (lane 4), and bovine serum albumin (lane 5) using rabbit anti-rVpmaX serum and a peroxidase-conjugated secondary antibody.

We used a prokaryotic expression system to obtain a soluble recombinant protein that migrated with a molecular mass of approximately 35 kDa on SDS-PAGE. This recombinant protein was used to raise specific rabbit polyclonal antiserum against *M. bovis* VpmaX. The serum specifically reacted with the 35 kDa band of rVpmaX (Figure 1, lane 1) and with a 30 kDa band from a *M. bovis* whole cell preparation (Figure 1, lane 2).

### Localization of *M. bovis* VpmaX

*M. bovis* VpmaX was detected in the cell membrane protein fraction (Figure 1, lane 3) and in the whole-cell proteins (Figure 1, lane 2). Recombinant VpmaX (Figure 1, lane 1) and bovine serum albumin (Figure 1, lane 5) served as positive and negative controls, respectively. This analysis, using an anti-rVpmaX antibody, revealed a clear interaction with a protein of approximately 30 kDa in the membrane fraction and whole-cell proteins, indicating that VpmaX existed in the membrane of *M. bovis* Hubei-1.

### rVpmaX Direct Adhesion to EBL Cells and Adhesion Inhibition Assay

**Figure 2:**
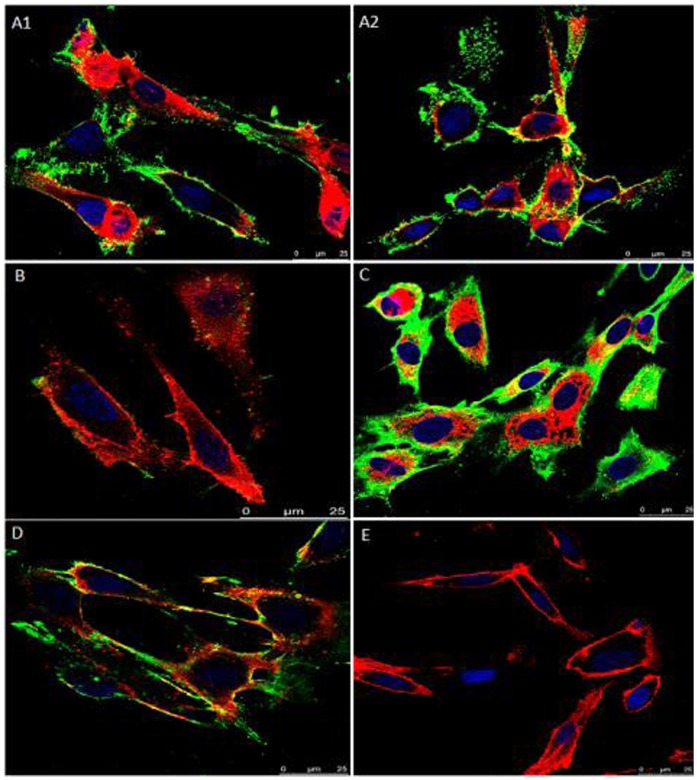
Assay of rVpmaX adhesion and adhesion inhibition to EBL cells visualized by confocal laser scanning microscopy. Active rVpmaX interacted with fixed EBL cells, and the surplus protein was rinsed away by washing with PBST. The attached protein was immunostained with rabbit anti-rVpmaX antibody and mouse anti-rabbit IgG-FITC. The EBL cell membranes were labeled with 1,19-dioctadecyl-3,3,3′,3′-tetramethylindocarbocyanine perchlorate (DiI), and the cell nuclei were counter-labeled with 49,6-diamidino-2-phenylindole (DAPI). (A1–A2) 10 µg rVpmaX adhering to EBL cells. (B) Adhesion inhibition of 10 µg rVpmaX to EBL cells by 10 µl rabbit anti-rVpmaX serum. (C) Adhesion of 20 µg rVpmaX to EBL cells. (D) The adhesion of 20 µg rVpmaX to EBL cells was inhibited by 20 µl rabbit anti-rVpmaX serum. (E) EBL cells without protein added.

The adhesion of rVpmaX to EBL cells was visualized by laser scanning confocal microscopy. As shown in Figure 2, we found that rVpmaX strongly adhered to EBL cells, and this adherence could be inhibited by rabbit anti-rVpmaX serum. When a small amount of rVpmaX was added to the EBL cells, the majority of the protein mainly attached to the cell membrane (Figure 2, A1–A2); the protein began to enter the cytoplasm as the protein amount increased (Figure 2C). Moreover, rVpmaX adherence to EBL cells was inhibited by rabbit anti-rVpmaX serum (Figure 2B, D). The cells with no protein added showed no fluorescence (Figure 2E). The results of confocal laser scanning microscopy (CLSM) therefore indicated that rVpmaX has a substantial ability to adhere to host cells.

### Quantitative Adhesion and Adhesion Inhibition Assay by Sandwich ELISA

**Figure 3:**
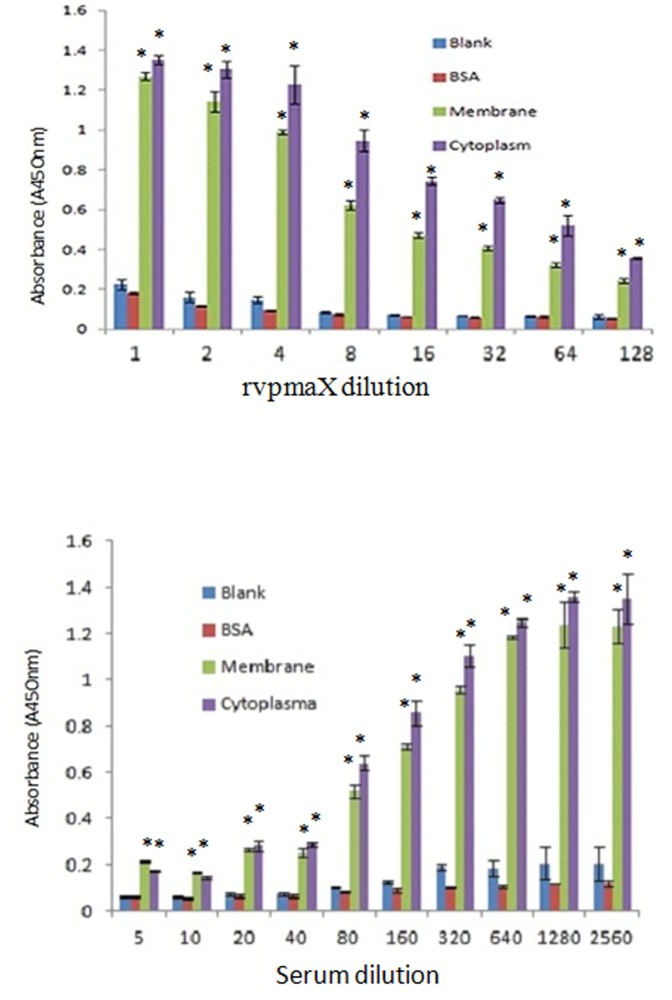
Sandwich ELISA tests of rVpmaX adhesion and adhesion inhibition. ELISA plates were coated with 1 µg EBL cell membrane or cytoplasmic fraction proteins per well. Plates coated with an equivalent amount of BSA and non-coated plates serve as the negative control and blank control, respectively. (A) 1 µg rVpmaX in 100 µl PBST was serially diluted to 128-fold and incubated with the treated plates. (B) The adhesion of 0.5 µg rVpmaX was inhibited by decreasing concentrations of anti-rVpmaX antiserum (in serial dilutions from 1/5 to 1/2560). *P<0.01, compared with the absorbance of BSA-coated wells in the corresponding group.

Different dilutions of rVpmaX were added to ELISA wells coated with EBL cell membrane fraction proteins. We found that the rVpmaX adherence indices (Figure 3A) decreased as the protein dilutions increased. A similar pattern was also observed when the plates were coated with equivalent amount of EBL cell soluble cytosolic fraction proteins (Figure 3A). In the adhesion inhibition test, a certian amount of rVpmaX adhesion was inhibited by different dilutions of the rabbit anti-rVpmaX antibody. The addition of increasing concentrations of anti-rVpmaX antibodies decreased rVpmaX binding to both the EBL membrane and cytosolic fraction proteins in a dose-dependent manner (Figure 3B). In both assays, blank wells and wells coated with an equivalent amount of BSA showed few interactions with rVpmaX (Figure 3A, B). A similar trend was also observed when rVpmaX was replaced with *M. bovis* membrane fraction proteins in another adhesion and adhesion inhibition assay. The sandwich ELISA results further validated that VpmaX mediates adhesion to EBL cells, and this conclusion is consistent with the CLSM results. Moreover, we found that the EBL cytosolic fraction proteins also can attach VpmaX, which may imply a role for *M. bovis* VpmaX in some pervasive reactions in EBL cells.

## Discussion

*M. bovis* is the causative agent of chronic pneumonia in feedlot cattle and dairy calves [3]. Due to the frustrating result of antibiotic therapy in its treatment, this organism causes substantial economic losses for the cattle industry worldwide. It has been fifty-two years since the identification of this microbe [1], but its pathogenesis is still unclear, mainly because the research techniques available for this organism are limited. Because adhesion to susceptible host cells is a prerequisite for colonization and infection for many bacteria, current research is focused on the adhesion component of mycoplasma. Vsps in the *M. bovis* strain PG45 was confirmed to be involved in *M. bovis* cytoadhesion to host cells [11], [17]. Moreover, as immunodominant lipoproteins in *M. bovis*, Vsps underwent high-frequency phase and size variations during natural infection [26] and have therefore attracted the most interest in *M. bovis* research.

The Vsps gene family has been reported to be a characteristic region in *M. bovis* [23] and was reported to be completely deleted in the *M. bovis* strain Hubei-1 [20]. However, our study identified a surface protein in *M. bovis* Hubei-1, the VpmaX, that contains some traits similar to those of the Vsp proteins in PG45. First, it has seven short repetitive units of four aa and three long repetitive sequences comprising sixteen aa. Research in *M. pulmonis* revealed that a long Vsa protein with many tandem repeats (35 or 40 repeats) contributed less to adhesion than a short Vsa containing fewer repeats (3, 4, or 5 repeats) [10]. Therefore, we hypothesized that VpmaX would have a substantial ability to adhere host cells. This was affirmed by the CLSM and sandwich ELISA assay. Second, although VpmaX possesses no conserved sequence in the N-terminal protein region and upstream DNA sequence like the Vsps family in PG45, it does have a typical lipoprotein signal sequence, which likely anchor it to the *M. bovis* membrane. The VpmaX localization assay confirmed our speculation that VpmaX existed in the membrane fragment of *M. bovis*. Finally, *M. bovis* VpmaX expressed in *E. coli* displayed an abnormal migration in SDS-PAGE gel: the rVpmaX molecular weights calculated from the deduced sequence is 28 kDa, but its molecular mass according to the position of the band after SDS-PAGE is approximately 35 kDa. A similar phenomenon was also observed in PG45 VspB [23], a protein of 268 aa with a predicted mass of 25 kDa that has an electrophoretic migration that suggests the product expressed in *E. coli* and in mycoplasma has a mass of 46 kDa.

A comparison between VpmaX and its counterpart in HB0801, VspX, shows that the sequences of the two proteins are identical. However, there is some variation between VpmaX and its corresponding protein in PG45, PL. This is also consistent with a previous study that high frequency of variation occurred within the products of the various *vsp* genes due to spontaneous insertions or deletions of repetitive units in *M. bovis* [23]. Because *M. bovis* Hubei-1 and HB0801 were both isolated in Hubei province, it is reasonable that the two strains contain identical protein sequences in VpmaX.

Many surface proteins in mycoplasma function as adhesions to host cells. Our study verified that recombinant VpmaX and natural VpmaX can strongly adhere to EBL cells using CLSM and sandwich ELISA. Previous research demonstrated that *M. bovis* Vsp antigens were constantly present in the cytoplasm of macrophages and were also present on the surfaces of epithelial cells in larger airways when cattle were experimentally infected with *M. bovis* strain 1067 [27]. We observed a similar distribution of rVpmaX in EBL cells. Figure 2C and Figure 3 indicate that rVpmaX adhesion is pervasive on the EBL membrane and in the cytoplasm. The widespread existence of proteins in EBL cells that VpmaX can adhere to implies that *M. bovis* VpmaX may relate to some critical and common mechanism when *M. bovis* evading host body.

The variability of mycoplasma surface antigens under immune pressure has been reported for several mycoplasma species [28], [29]. For example, the expression of the protein pMGA in *Mycoplasma gallisepticum* ceased when grown in broth containing an antibody against pMGA, and the organism instead expressed an antigenically unrelated new polypeptide (p82) [29]. We also have done some preliminary works to examine the antigenic variation of VpmaX. However, our immune pressure test [28] showed that *M. bovis* grown in broth containing anti-rVpmaX serum still expressed VpmaX, but after the fifth passage under immune pressure, the organism expressed a new protein with a molecular mass of 55 kDa that reacted with the rabbit anti-rVpmaX serum. The isolation and identification of this newly observed 55 kDa protein using mass spectroscopy is still in progress. However, the persistent expression of VpmaX under immune pressure may indicate that this protein may plays an important role in the survival of *M. bovis* strain Hubei-1. This speculation can also be supported by the prevalence of VpmaX interacting proteins in EBL cells.

### Conclusion

In this study, we characterized various aspects of the *M. bovis* Hubei-1 protein VpmaX, including the existence of repetitive units, its localization in the membrane, unusual migration in SDS-PAGE; these features are shared with Vsps in PG45 and HB0801. Furthermore, VpmaX has been confirmed to be an adhesion-related protein that is able to strongly adhere to EBL cells. The pervasive VpmaX adhesion in EBL cells suggests that this protein may play a role in some important process during *M. bovis* infection, which is worth further exploration. Understanding the interplay between adhesion proteins and host cells will allow for a better understanding of *M. bovis* infection and may facilitate its prevention.
